# A Case,—Its Treatment

**Published:** 1865

**Authors:** S. Driggs


### A CASE.—ITS TREATMENT.

BY S. DRIGGS.

**Read before the Mississippi Valley Dental Association.**

As the subject of the restoration of the loss of the bony structure of the mouth has been attracting considerable attention of late years, I beg leave to present the following case:

About the middle of August last, a gentleman thirty years of age, billious temperament, and apparently good constitution, presented himself for consultation and treatment. Pus was issuing from the nostril in profuse quantities, the mucous membrane of the left side of the palatine vault was largely distended, and the nerve of the superior left lateral incisor was dead. Upon raising the lip, a slight cicatrix showed that pus had previously found exit at that point. Not suspecting the extent of the disease, I introduced a lancet into the tumor of the palatine arch to allow the large quantity of pus collected there to escape; but, to my astonishment, the instrument, after passing through the mucous membrane, met with no resistance. To ascertain clearly the extent of disease, I passed a probe through the incision, through the palate bone, into the maxillary sinus; from thence through the fistulous canal, and out at its orifice on the labial side of the alveolus, between the lateral incisor and canine. Absorption of bone had resulted in an *aperture* through the palatine arch, in size about three lines in width by fifteen in length.

TREATMENT.—After copious injections of warm water, a tent of cotton was placed in the incision, and the patient dismissed for the day. The cotton was removed every alternate day for two weeks, and the cavity thoroughly cleansed, and a solution of sulphate of zinc injected. During that time, I had endeavored to accomplish nothing more than lessening the quantity of purulent matter.

In the meantime, the propriety, or impropriety, of extracting the offending tooth had been considered, and I determined to make a vigorous effort for its salvation. The crown was well preserved and the color good; and I could not reconcile it with my sense of duty to my patient and to the profession to make it a "military necessity" and pass the sentence of banishment upon it without a fair and patient trial. Writers upon the subject have, I know, invariably condemned such offenders to the tender mercies of the forceps; but I have not always found it best to follow blindly rules which are supposed to have no exceptions.

An old plug was now removed from the anterior approximal surface, and nerve canal and crown plugged in the usual manner. The seton was removed from the incision before mentioned, and the membrane allowed to heal. The fistulous orifice between the lateral incisor and canine was enlarged to the full distance between them, and all subsequent applications introduced thereat. Injections of tepid water and dressings of lint, saturated with dilute creosote, were applied as often as the patient could conveniently attend, which, as he resided eight miles in the country, and was engaged in business which called him much from home, was sometimes twice and sometimes once a week. This treatment, with an occasional change to iodine, having been continued for a month without satisfactorily changing the character of the discharge, my convictions as to the existence of at least mercurio-syphilitic taint were strengthened, and iodide of potassium, in comp. sirup of sarsaparilla, was prescribed, and the creosote pushed (as Dr. Atkinson would say) "heroically."

Decided manifestations of improvement soon became apparent; but the patient, beginning to complain also of great circum-orbital pain, it was considered as a remonstrance against the prolongation of such heroic treatment, and milder applications, such as injections of tincture of arnica and glycerine *a a* were found beneficial, and have been continued, with an occasional stimulus from very dilute creosote, to the present time.

Healthy granulations were rapidly formed, and ossification has taken place, to the extent of the complete restoration of the palate bone.

My attention is now being directed to the restoration of the lost alveolar substance, which, I think, will be speedily effected.

It is a source of gratification to myself, as well as to the patient, that the diseased tooth was not extracted. It has not prevented, although it may have retarded, the cure. It is not always well "to do evil that good may come." A little patience, a little energy, and considerable perseverance, will sometimes enable us to accomplish what we are accustomed to regard as impracticable.

In conclusion, gentlemen, I acknowledge that I have groped my way along with but a dim conception and a beggarly knowledge of appropriate remedies, the manner of their application, their "modus operandi," or the exact results to be expected. You will, therefore, pardon any faults which a better knowledge and a larger experience have enabled you to discern.

